# Awareness and Attitude Toward Artificial Intelligence Among Medical Students and Pathology Trainees: Survey Study

**DOI:** 10.2196/62669

**Published:** 2025-01-10

**Authors:** Anwar Rjoop, Mohammad Al-Qudah, Raja Alkhasawneh, Nesreen Bataineh, Maram Abdaljaleel, Moayad A Rjoub, Mustafa Alkhateeb, Mohammad Abdelraheem, Salem Al-Omari, Omar Bani-Mari, Anas Alkabalan, Saoud Altulaih, Iyad Rjoub, Rula Alshimi

**Affiliations:** 1 Department of Pathology and Microbiology, Faculty of Medicine, Jordan University of Science and Technology, Irbid, 22110, Jordan, 962 796958408, 962 2 7095123; 2 Department of Microbiology, Pathology and Forensic Medicine, Faculty of Medicine, The Hashemite University, Zarqa, Jordan; 3 Department of Pulmonary Medicine, King Hussain Medical Center, Royal Medical Services, Amman, Jordan; 4 Department of Basic Medical Sciences, Faculty of Medicine, Yarmouk University, Irbid, Jordan; 5 Department of Pathology, Microbiology, and Forensic Medicine, School of Medicine, The University of Jordan, Amman, Jordan; 6 Department of General Surgery and Urology, Faculty of Medicine, Jordan University of Science and Technology, Irbid, Jordan; 7 Faculty of Medicine, Jordan University for Science and Technology, Irbid, Jordan

**Keywords:** artificial intelligence, AI, deep learning, medical schools, pathology, Jordan, medical education, awareness, attitude, medical students, pathology trainees, national survey study, medical practice, training, web-based survey, survey, questionnaire

## Abstract

**Background:**

Artificial intelligence (AI) is set to shape the future of medical practice. The perspective and understanding of medical students are critical for guiding the development of educational curricula and training.

**Objective:**

This study aims to assess and compare medical AI-related attitudes among medical students in general medicine and in one of the visually oriented fields (pathology), along with illuminating their anticipated role of AI in the rapidly evolving landscape of AI-enhanced health care.

**Methods:**

This was a cross-sectional study that used a web-based survey composed of a closed-ended questionnaire. The survey addressed medical students at all educational levels across the 5 public medical schools, along with pathology residents in 4 residency programs in Jordan.

**Results:**

A total of 394 respondents participated (328 medical students and 66 pathology residents). The majority of respondents (272/394, 69%) were already aware of AI and deep learning in medicine, mainly relying on websites for information on AI, while only 14% (56/394) were aware of AI through medical schools. There was a statistically significant difference in awareness among respondents who consider themselves tech experts compared with those who do not (*P*=.03). More than half of the respondents believed that AI could be used to diagnose diseases automatically (213/394, 54.1% agreement), with medical students agreeing more than pathology residents (*P*=.04). However, more than one-third expressed fear about recent AI developments (167/394, 42.4% agreed). Two-thirds of respondents disagreed that their medical schools had educated them about AI and its potential use (261/394, 66.2% disagreed), while 46.2% (182/394) expressed interest in learning about AI in medicine. In terms of pathology-specific questions, 75.4% (297/394) agreed that AI could be used to identify pathologies in slide examinations automatically. There was a significant difference between medical students and pathology residents in their agreement (*P*=.001). Overall, medical students and pathology trainees had similar responses.

**Conclusions:**

AI education should be introduced into medical school curricula to improve medical students’ understanding and attitudes. Students agreed that they need to learn about AI’s applications, potential hazards, and legal and ethical implications. This is the first study to analyze medical students’ views and awareness of AI in Jordan, as well as the first to include pathology residents’ perspectives. The findings are consistent with earlier research internationally. In comparison with prior research, these attitudes are similar in low-income and industrialized countries, highlighting the need for a global strategy to introduce AI instruction to medical students everywhere in this era of rapidly expanding technology.

## Introduction

Artificial intelligence (AI) is the capability of machines to simulate intelligence by exhibiting human-like traits such as understanding, deductive reasoning, and problem-solving abilities [1]. In medicine, many specialties have already used AI in clinical practice, such as oncology for cancer detection and grading [2]. AI, particularly deep learning, has garnered a lot of attention in medical education and pathology in recent years [3,4]. These techniques have been mostly used for visual activities such as consultations, seminar presentations, board exams, and archiving [5]. Even before this achievement, many experts predicted that specialized algorithms capable of reading images as well as or better than human observers would dominate the future of medicine. As a result, residents and undergraduate students are becoming increasingly concerned that pursuing medicine training may be an insecure career path [6]. However, the long-awaited expansion of AI in pathology is still underway, and the area of pathology is changing at a far slower rate than other domains (eg, radiology) [7].

The recent approvals of whole slides imaging scanners by the Food and Drug Administration for primary diagnosis, as well as the approval of the prostate AI algorithm, have cleared the road for the first steps in incorporating this new technology for use in pathologic primary diagnosis. AI solutions can serve as a unique platform for breakthroughs and advancements in anatomical and clinical pathology practice [8].

Commercially available AI systems have recently become the focus of a more concentrated evaluation. However, it is unclear whether medical students and pathology residents are afraid that AI may replace pathologists or other clinicians [9]. There is limited knowledge about medical students’ perspectives toward AI and deep learning, particularly in underdeveloped nations. To investigate this topic, we conducted a multicenter national survey of undergraduate medical students and pathology residents to assess their perceptions of AI in medicine in general and pathology in particular, as well as their concern that machines will replace pathologists or other physicians soon.

The perspective and understanding of medical students are critical for guiding the development of educational curricula and training. We investigate major medical AI-related attitudes among medical students in general and in pathology in Jordan, with an emphasis on the components that should be included in the medical curriculum.

## Methods

### Study Design and Population

A randomized, web-based, cross-sectional study was conducted among medical students and pathology residents in Jordan from 5 public universities—Jordan University of Science and Technology (JUST), The University of Jordan, Yarmouk University, Hashemite University, and Mutah University, including about 19,000 medical students—in addition to 4 pathology residency programs: JUST, King Hussein Cancer Center, Jordan University Hospital, and Royal Medical Services, including about 80 pathology residents. The survey was published over a 10-day period from March 4 to March 14, 2024. The required sample size was estimated using the web-based Raosoft sample size calculator [10]. The proposed sample size was 318, with a 5% margin of error, a 99% confidence, a 30% response rate, and a population size of 20,000. Three hundred ninety-four students completed the questionnaire. Students were encouraged to disseminate the questionnaire among their colleagues to create a snowball sample.

### Ethical Considerations

The research has been approved by the institutional review board of JUST, Irbid, Jordan (number: 3/167/2024; date: February 13, 2024). All individuals consented to participate. All methods met with the applicable standards and regulations. Participants provided informed consent at the beginning of the survey and had the ability to opt out at any time. Data were anonymized and no compensation was provided to the participants. The results of this study are original, have not been previously published, are not under review elsewhere, and have received approval from all authors. All the authors have approved final version of the manuscript.

The data were obtained using a self-administered open web-based questionnaire produced with Google Forms. To evaluate the questionnaire’s applicability and forward validity, our research team translated the questions into Arabic and conducted a pilot survey with 15 randomly selected participants to examine question comprehension and language clarity. The questionnaire was circulated through medical student groups, social media forums (Facebook, WhatsApp, Telegram, and Instagram), and through announcements in lectures. Participation was entirely voluntary and unrelated to the students’ educational curriculum. The students consented to participate by completing the survey. Respondent anonymity was assured by design.

### Questionnaire Structure

The questionnaire items were adopted from a previously validated study [11] and amended by 2 expert pathologists (AR and MA) at 2 academic centers to apply the questions to the discipline of pathology. The questionnaire was divided into sections, each of which addressed a different issue (Table S1 in Multimedia Appendix 1). The first section of the questionnaire inquired about demographic data and self-reported technological expertise. The second portion inquired about AI and deep learning applications in medicine. The third part evaluated sources to AI in general. The fourth part focused on AI applications in medicine. The fifth section evaluated emotions and perspectives toward AI and deep learning in medicine and pathology. The sixth part inquired about the expected effects of AI on medical education and specific components that should be implemented in medical education (an open-ended question), followed by a question regarding whether basic AI knowledge should be provided in official medical courses (a Yes/No). Finally, the prospective applications of AI in pathology were considered.

### Statistical Analysis

Following the completion of questionnaire submissions, the findings were converted to a comma-separated value file. To simplify statistical analysis, the categories “disagree entirely” and “rather disagree” were summarized as disagreement, while “rather agree” and “agree entirely” were summarized as agreement. Nominal categorical variables were analyzed using the Pearson chi-square test or Fisher exact test, whereas ordinal data were analyzed using Spearman correlation. The statistical analysis was performed using SPSS version 26.0 (IBM Corp) [12], and *P* values of <.05 were considered statistically significant.

## Results

### Overview

After 10 days of opening the survey, 394 participants completed the questionnaire (328 medical students and 66 pathology residents). Of these respondents, 49% (193/394) were males and 51% (201/394) were females. The median age is 20 (IQR 20‐21) years. Most medical students surveyed were in their junior years (1, 2, and 3), accounting for around 85% (279/394) of the sample, while approximately 15% (49/394) were in clinical training years (4, 5, and 6). Most pathology residents surveyed were postgraduate year 1 and postgraduate year 2 (63/66, 96%). Of the total, 44.9% (177/394) of the respondents regarded themselves as technological experts (Table 1).

**Table 1.** Demographics and self-reported technical expertise (N=394).

		Participants
I consider myself a tech expert person, n/N (%)		
	Agree entirely	46/394 (12)
	Rather agree	131/394 (33.2)
	Rather disagree	73/394 (19)
	Disagree entirely	16/394 (4)
	—^a^	128/394 (32.5)
Age (years)		
	Median	20
	IQR	20-21
	Minimum/maximum	18/34
Gender, n/N (%)		
	Male	193/394 (49)
	Female	201/394 (51)

a Not available (or no response).

### Awareness of AI and Deep Learning in Medicine

The vast majority of respondents (272/394, 69%) were previously aware of the medical community’s discussion on AI and deep learning. There was a statistically significant difference in awareness among respondents who consider themselves tech experts compared with those who do not (*P*=.03), but no significant differences were found between males and females, medical students, and pathology residents. Furthermore, 67.5% (266/394) of respondents reported having a basic awareness of the technologies used in these fields (Table 2).

**Table 2.** First part of the questionnaire—artificial intelligence and deep learning in medicine (N=394).

Questionnaire items		Yes, n/N (%)	No, n/N (%)	*P* value^a^	
“Deep learning” and “artificial intelligence” are currently being broadly discussed in the medical field.					
	Were you already aware of these topics?	272/394 (69)	122/394 (31)	.03/.46	
	Do you have a basic understanding of the technologies used in these topics?	266/394 (67.5)	128/394 (32.5)	.89/.94	

a Tech expert versus non–tech expert/medical students versus pathology residents.

### The Sources for the Topic of AI

Websites were the primary sources of knowledge on AI, with 73.4% (289/394) of respondents reporting awareness via this source. In contrast, fewer students heard about AI from friends and colleagues (139/394, 35.3%), webinars (59/394, 15%), and medical school lectures (56/394, 14%). The majority of respondents (310/394, 78.6%) acknowledged that they did not learn about AI through formal AI courses (Table 3).

**Table 3.** Second part of the questionnaire—different sources of exposure to artificial intelligence as a topic in general (N=394).

Questionnaire items		Yes, n/N (%)	No, n/N (%)	*P* value^a^
Other applications we use in daily life already use artificial intelligence (eg, speech-/text-recognition). Were you aware of this from?				
	Websites	289/394 (73.4)	105/394 (26.6)	<.001/.66
	Social friends and colleagues	139/394 (35.3)	255/394 (64.7)	.20/.74
	Medical school lectures	56/394 (14)	338/394 (85.8)	.90/.28
	Webinars	59/394 (15)	335/394 (85)	.80/.37
	Training (eg, courses) in artificial intelligence	22/394 (6)	372/394 (94.4)	.15/.99
	No answer	—^b^	—	.001/.85

a Tech expert vs non–tech expert/medical students vs pathology residents.

b Not applicable (ie, total proportion of no answers: 62/394, 16%).

### The Applications of AI in Medicine

More than half of respondents thought that AI might be used to diagnose disease in patients automatically (213/394, 54.1% agreement vs 82/394, 21% disagreement), and medical students agreed more than pathology residents on this topic *(P*=.04). Furthermore, 39.5% (156/394) agreed to the use of AI in automated diagnosis. Moreover, three-quarters agreed that AI might automatically indicate appropriate investigations (318/394, 80.7% agreement vs 19/394, 5% disagreement). Table 4 provides more detailed results.

**Table 4.** Third part of the questionnaire—applications for artificial intelligence in medicine (N=394).

Questionnaire items		Agree entirely, n/N (%)	Rather agree, n/N (%)	Rather disagree, n/N (%)	Disagree entirely, n/N (%)	N/A^a^, n/N (%)	*P* value^b^
What potential applications for AI in medicine do you see?							
	Automated detection of disease	55/394 (14)	158/394 (40.1)	69/394 (16)	13/394 (3)	99/394 (25)	.58/.001
	Automated diagnosis of patients	39/394 (10)	117/394 (29.7)	107/394 (27.2)	19/394 (5)	112/394 (28)	.016/.000
	Automated indication of appropriate investigations (radiological, laboratory, etc)	113/394 (28.7)	205/394 (52)	17/394 (4)	2/394 (1)	57/394 (15)	<.001/.60

a N/A: not applicable.

b Tech expert vs non–tech expert/medical students vs pathology residents.

### Overall Emotions and Attitudes About AI and Deep Learning in Both Medicine and Pathology

Regarding overall feelings and attitudes toward AI and deep learning in medicine and pathology, most respondents agreed that AI will revolutionize medicine in general (321/394, 81.4% agreement) and pathology in particular (312/394, 79.2% agreement), while a sizable proportion disagreed that human doctors in general (291/394, 73.9% disagreement) and pathologists (248/394, 63% disagreement) could be replaced in the near term. Furthermore, more than one-third of respondents expressed fear about recent AI developments (167/394, 42.4% agreed).

On the other hand, roughly half said that these breakthroughs make pathology or medicine more intriguing to them (184/394, 46.7% and 222/394, 56.4%, respectively), and for those specific questions, tech expert respondents were considerably more likely to say “yes” than non–tech expert respondents (*P*=.002 and *P*=.03, respectively).

Nonetheless, most respondents believed that the adoption of AI would benefit pathology (302/394, 76.7% agreement) and the entire field of medicine (305/394, 77.4% agreement). Notably, two-thirds of respondents disagreed that their medical schools or hospitals had educated them about AI and its uses (261/394, 66.2% disagreed), whereas 46.2% (182/394) expressed an interest in learning the principles of AI and its applications in medicine. Table S1 in Multimedia Appendix 1 summarizes feelings and attitudes toward AI and deep learning in medicine and pathology.

### What Specific Aspects Should Be Implemented in Medical Education?

Students were asked to select from a list of options, each of which allowed for several responses. The majority of respondents (231/394, 58.6%) expressed an interest in learning about AI’s applications, potential hazards, and legal and ethical implications. Females were more likely than males to exhibit an interest in learning about AI’s possible hazards and legal implications (*P*=.038 and *P*=.001, respectively). In addition, 51.8% (204/394) of the students felt that medical education should cover current AI systems and their technical foundations. However, 64.2% (253/394) of respondents expressed no interest in learning about the classification of AI reliability in medical education (Table 5).

**Table 5.** Specific aspects that should be implemented in medical education (multiple responses possible) (N=394)^a^.

	Yes, n/N (%)	No, n/N (%)	*P* value^b^
Areas of application	231/394 (58.6)	163/394 (41.4)	.04/.95
Possible risks	231/394 (58.6)	163/394 (41.4)	.25/.83
Technical basics	204/394 (51.8)	190/394 (48.2)	.73/.74
Current AI^c^ systems	204/394 (51.8)	190/394 (48.2)	.63/.52
Modes of operation	200/394 (50.8)	194/394 (49.2)	.30/.21
Legal aspects, ethics	198/394 (50.3)	196/394 (49.7)	.094/.35
Potential future developments	195/394 (49.5)	199/394 (51.5)	.45/.36
Classification of AI reliability	141/394 (35.8)	253/394 (64.2)	.77/.23
Basic AI knowledge should be provided in university courses	357/394 (90.6)	37/394 (9.4)	.21/.16

a The vast majority of respondents believed that university curricula should cover fundamental AI concepts (357/394, 90.6% said yes).

b Tech expert vs non–tech expert/medical students versus pathology residents.

c AI: artificial intelligence.

### What Potential Applications for AI Do You See in Pathology?

In terms of pathology-specific questions, 3 quadrants (297/394, 75.4%) of respondents agreed that AI could be used to identify pathologies in slides examinations automatically, and more than half (222/394, 56.3%) agreed that AI could be used to diagnose pathologies in slides examinations and indicate appropriate further stains needed. There was a statistically significant difference between medical students, who are more inclined to agree, and pathology residents (*P*=.02/*P*<.001 and *P*<.001/*P*=.61, respectively). In addition, 79.1% (312/394) of respondents felt that AI might be used to automatically identify appropriate special studies and immunohistochemistry stains in slide examinations (Table 6).

**Table 6.** Potential applications for artificial intelligence in pathology (N=394).

	Totally agree, n/N (%)	Agree, n/N (%)	Disagree, n/N (%)	Totally disagree, n/N (%)	Neutral, n/N (%)	*P* value^a^
Automated detection of pathologies in slides examinations	91/394 (23)	206/394 (52.3)	26/394 (7)	5/394 (1)	66/394 (17)	.56/.001
Automated diagnosis in slides examinations	58/394 (15)	164/394 (41.6)	50/394 (13)	11/394 (3)	111/394 (28.2)	.02/<.001
Automated indication of appropriate special studies and immunohistochemical stains in slides examinations	103/394 (26.1)	209/394 (53)	11/394 (3)	2/394 (1)	69/394 (18)	<.001/.61

a Tech expert vs non–tech expert/medical students vs pathology residents.

## Discussion

### Principal Findings

Findings revealed that a significant majority of medical students were already aware of the ongoing discussion around AI and deep learning in the medical community. This awareness was significantly higher among those who described themselves as tech savvy. The survey results support the conclusion that the majority of respondents did not learn about AI throughout medical school.

The majority of respondents thought that AI had the potential to alter both medicine in general and pathology in particular. However, a significant proportion raised concerns about the potential displacement of human physicians by AI in the near future. This sentiment is consistent with other research revealing worries among medical students and pathology residents regarding the expanding role of AI in medicine. For example, in Lebanon and Kuwait, there was an agreement that AI would not replace doctors but rather significantly transform health care practices [13,14]. Notably, the majority of medical students who responded were in their junior years (279/394, 85%) of the total sample, which may reflect their interest in the subject compared with senior students. Although this allowed us to compare junior medical students’ perceptions with those of postgraduate pathology students, it is regarded as a restriction for evaluating senior medical students’ perspectives.

The general agreement was that the use of AI would benefit both pathology and the larger field of medicine [8]. Further comparison with prior work is discussed in the section “Comparison With Prior Work.” This study provides useful insights into medical students’ perspectives and attitudes regarding AI, which are critical for guiding the development of educational curricula and training.

The rapid growth of AI has the ability to change the face of a variety of medical specialties. Specifically in the visual medical disciplines, such as radiology, pathology, ophthalmology, and dermatology, AI has generated significant interest and will particularly affect the developments of these fields due to the visual nature of their occupations [3]. Deep learning applications are autonomously trained to execute certain tasks in response to the availability of large digital datasets. Visual activities include consultations, seminar presentations, board exams, and archiving [5]. Medical students’ perspectives are crucial for shaping medical education, especially in rapidly changing professions. This study collects students’ opinions on AI in medicine in order to better understand their requirements and expectations from medical schools, as well as add to the dataset so that data from various countries (both low and high income) may be compared to analyze future medical education plans. Learners in the digital era differ from past generations. They are growing increasingly technologically savvy and socially conscious. Pathology and radiology are 2 visual fields that have seen significant advancements in AI technology. Many studies have been conducted in radiology [11], but to our knowledge, this is the first to analyze the attitudes and awareness of pathology residents.

### AI-Related Attitudes of Medical Students in Comparison With Pathology Residency Trainees

To the best of our knowledge, this is the first study to discuss pathology residents’ awareness and attitudes toward AI in the field of pathology, in addition to comparing it with medical students’ perspectives. In Jordan, digital pathology is a relatively new concept that is primarily used for research purposes. A digital scanner is available at one site in Jordan (at JUST), with a focus on research and consulting purposes.

In general, there was no major difference in the responses between undergraduate students and postgraduate pathology residents. Except on 2 occasions, medical students agreed more than pathology residents that AI can be used to diagnose disease in patients automatically (*P*=.04). There was also a statistically significant difference between medical students, who are more likely to agree than pathology residents regarding AI’s ability to diagnose pathologies in slide examinations and indicate appropriate additional stains needed (*P*=.02/*P*<.001 and *P*<.001/*P*=.61, respectively). The Food and Drug Administration’s recent support of whole-slide imaging scanners for primary diagnosis, together with the approval of prostate AI algorithms, marks the beginning of introducing AI technology into primary diagnostics. AI can provide a unique platform for promoting innovation and breakthroughs in anatomical and clinical pathology practice [9].

Otherwise, there was general agreement between medical students and pathology residents that, for example, the adoption of AI would benefit pathology and the entire field of medicine and that they needed to learn about AI’s applications, potential hazards, and legal and ethical implications.

In summary, the findings underscore the imperative to integrate AI education into the medical field to adeptly equip future physicians for AI-augmented health care. Subsequent investigations should concentrate on assessing the efficacy of embedding AI education into medical training programs, both undergraduate and postgraduate, and probing the determinants influencing medical students’ attitudes toward AI. AI may also be used in pathology to diagnose cancer, predict survival, modify molecular structures, and forecast treatments. More effort is required to navigate these applications. Furthermore, it is worth investigating the relationship between the extent of digital pathology implementation and AI awareness.

### Limitations

Some limitations include the sample strategy’s use of social media as well as the web-based approach, which limits randomization and generalization. Also, the majority of medical students who responded were in their junior years (279/394, 85%). Although this allowed us to compare junior medical students’ perceptions to those of postgraduate pathology students, it is regarded as a restriction for evaluating senior medical students’ perspectives.

### Comparison With Prior Work

Compared with published work in this regard, similar results were identified in neighboring countries, including Lebanon [13]. In Kuwait, there was also an agreement that AI would not replace doctors but rather significantly transform health care practices [14]. In the United Arab Emirates, there was a lack of acquaintance with AI found in a study that called for the introduction of specific education and training in medical schools [15]. In addition, identical outcomes were observed in industrialized countries. In Germany, two-thirds of students (539/838, 64.4%) believed that they were not well informed on AI in medicine, and 57.4% (463/807) thought that AI has beneficial applications in medicine, such as drug research, but less so for clinical use [16]. In the United States, a study showed that 91% (353/387) reported receiving no formal education related to AI [17].

### Conclusions

Our study highlights a generally favorable attitude toward AI between medical students and pathology residents. A significant number of participants are already familiar with the ideas of AI and deep learning in medicine while the majority sees potential in AI for automated detection of pathologies and indication of appropriate investigations, which is a warning about replacing physicians and pathologists in the nearest future.

The study found that being a tech expert influenced respondents’ awareness and attitudes toward AI. This indicates a potential gap in the current medical education system, with a large proportion of respondents expressing interest in learning more about AI and its applications in medicine.

In pathology, there is a prominent agreement on the potential applications of AI, particularly in the automated detection of pathologies in slide examinations and the automated indication of appropriate special studies and immunohistochemical stains. Medical students show that they are more enthusiastic about the integration of AI in pathology than pathology residents.

While most medical students and pathology residents acknowledge the potential of AI to revolutionize medicine and improve the pathology field, there is a clear need for integrating AI education into medical curricula and addressing concerns about its ethical and legal aspects.

## Supplementary material

10.2196/62669 Multimedia Appendix 1 Fourth part of the questionnaire—feelings and attitudes toward artificial intelligence and deep learning in medicine and pathology (N=394).

## Abbreviations

AI: artificial intelligence
JUST: Jordan University of Science and Technology
