# Supplementary material for: Awareness and Attitude Toward Artificial Intelligence Among Medical Students and Pathology Trainees: Survey Study
Source: JMIR Med Educ. 2025 Jan 10;11:e62669. doi: 10.2196/62669 (PMC11741511; doi:10.2196/62669)
Supplement: Multimedia Appendix 1 [file mededu-v11-e62669-s001.docx]

| **Multimedia Appendix 1.** Fourth part of the questionnaire – feelings and attitudes towards AI and deep learning in medicine and pathology (N= 394) | | | | | | |
| --- | --- | --- | --- | --- | --- | --- |
| In your personal opinion, how accurate are the following statements? | | | | | | |
|  | Agree entirely  (n, %) | Rather agree  (n, %) | Rather disagree  (n, %) | Disagree entirely  (n, %) | N/A  (n, %) | p-values (tech-expert vs. non-tech-expert/ medical students vs. pathology residents) |
| Artificial intelligence will revolutionize medicine in general | 125/394 (31.7%) | 196/394 (49.7%) | 24/394 (6%) | 1/394 (0.3%) | 48/394 (12%) | .09 / .04 |
| Artificial intelligence will revolutionize pathology | 112/394 (28.4%) | 200/394 (50.8%) | 18/394 (5%) | 2/394 (1%) | 62/394 (16%) | .014 / .59 |
| The human pathologist will be replaced in the near future | 27/394 (7%) | 53/394 (14%) | 152/394 (38.6%) | 96/394 (24%) | 66/394 (17%) | .08 / .78 |
| In the near future all physicians will be replaced | 24/394 (6%) | 37/394 (9%) | 111/394 (28.2%) | 180/394 (45.7%) | 42/394 (11%) | .84 / .09 |
| These developments frighten me | 66/394 (17%) | 101/394 (25.6%) | 74/394 (19%) | 39/394 (10%) | 114/394 (28.9%) | .39 / .20 |
| These developments make pathology more exciting to me | 52/394 (13%) | 132/394 (33.5%) | 47/394 (12%) | 17/394 (4%) | 146/394 (37.1%) | .002 / .37 |
| These developments make medicine in general more exciting to me | 57/394 (15%) | 165/394 (41.9%) | 46/394 (12%) | 10/394 (3%) | 116/394 (29.4%) | .03 / .32 |
| Artificial intelligence will improve pathology | 80/394 (20%) | 222/394 (56.3%) | 15/394 (4%) | 3/394 (1%) | 74/394 (19%) | .03 / .16 |
| Artificial intelligence will improve medicine in general | 98/394 (25%) | 207/394 (52.5%) | 12/394 (3%) | 4/394 (1%) | 73/394 (19%) | .03 / .79 |
| Your medical school or hospital taught you about Artificial Intelligence and its applications | 25/394 (6%) | 45/394 (11%) | 140/394 (35.5%) | 121/394 (30.7%) | 63/394 (16%) | .004 / .64 |
| You feel interested about learning the principles of Artificial Intelligence and its applications in medicine | 106/394 (26.9%) | 76/394 (19%) | 25/394 (6%) | 8/394 (2%) | 79/394 (20%) | .005 / .03 |
